# Supplementary material for: Inequality in electricity consumption and economic growth: Evidence from a small area estimation study
Source: PLoS One. 2023 Jul 26;18(7):e0284055. doi: 10.1371/journal.pone.0284055 (PMC10370772; doi:10.1371/journal.pone.0284055)
Supplement: S1 Table — (DOCX) [file pone.0284055.s002.docx]

Table A.1: GLS regressions of log of monthly per capita kWh: Northern Mountains

| Explanatory variables | Coefficient | Std. Err. | t | \|Prob\|>t |
| --- | --- | --- | --- | --- |
| Intercept | 1.749 | 0.307 | 5.694 | 0.000 |
| Having television (yes=1; no=0) | 0.694 | 0.061 | 11.332 | 0.000 |
| Commune proportion of households having television | 0.784 | 0.155 | 5.048 | 0.000 |
| Commune proportion of household head with primary school | -0.368 | 0.188 | -1.963 | 0.050 |
| Ethnic minorities (yes=1; no=0) | -0.291 | 0.052 | -5.564 | 0.000 |
| Household size | -0.103 | 0.015 | -7.000 | 0.000 |
| Average household size of commune | -0.133 | 0.049 | -2.733 | 0.006 |
| Log of per capita living area | 0.204 | 0.042 | 4.873 | 0.000 |
| Proportion of household members with upper-secondary school and above | 0.691 | 0.079 | 8.708 | 0.000 |
| Proportion of household members with lower-secondary school | 0.269 | 0.075 | 3.602 | 0.000 |
| Having house with solid roof (yes=1; no=0) | 0.158 | 0.054 | 2.928 | 0.004 |
| Using unclean water (yes=1; no=0) | -0.234 | 0.051 | -4.624 | 0.000 |
| Urban * Proportion of children in household | 0.606 | 0.184 | 3.290 | 0.001 |
| Urban * House with temporary wall | -0.510 | 0.208 | -2.459 | 0.014 |
| Number of observations | 1445 |  |  |  |
| R2-adjusted | 0.594 |  |  |  |
| Rho | 0.124 |  |  |  |

Notes: the estimation results are obtained from using data contained in the 2009 VPHC and the 2010 VHLSS
