# Supplementary material for: Inequality in electricity consumption and economic growth: Evidence from a small area estimation study
Source: PLoS One. 2023 Jul 26;18(7):e0284055. doi: 10.1371/journal.pone.0284055 (PMC10370772; doi:10.1371/journal.pone.0284055)
Supplement: S2 Table — (DOCX) [file pone.0284055.s003.docx]

Table A.2: GLS regressions of log of monthly per capita kWh: Red River Delta

| Explanatory variables | Coefficient | Std. Err. | t | \|Prob\|>t |
| --- | --- | --- | --- | --- |
| Intercept | 2.656 | 0.237 | 11.222 | 0.000 |
| Commune proportion of households having computer | 0.753 | 0.199 | 3.783 | 0.000 |
| Commune proportion of households having fridge | 0.914 | 0.132 | 6.909 | 0.000 |
| Having television (yes=1; no=0) | 0.271 | 0.063 | 4.277 | 0.000 |
| Log of the number of firms in commune | 0.039 | 0.018 | 2.179 | 0.030 |
| Log of household size | -0.224 | 0.043 | -5.267 | 0.000 |
| Log of per capita living area | 0.299 | 0.027 | 10.984 | 0.000 |
| Commune average of log of per capita living area | -0.157 | 0.077 | -2.035 | 0.042 |
| Proportion of children in household | 0.240 | 0.091 | 2.632 | 0.009 |
| Proportion of household households without primary school | -0.503 | 0.071 | -7.130 | 0.000 |
| Proportion of household members with lower-secondary school | -0.166 | 0.060 | -2.771 | 0.006 |
| Having house with solid roof (yes=1; no=0) | -0.286 | 0.112 | -2.550 | 0.011 |
| Number of observations | 1980 |  |  |  |
| R2-adjusted | 0.473 |  |  |  |
| Rho | 0.067 |  |  |  |

Notes: the estimation results are obtained from using data contained in the 2009 VPHC and the 2010 VHLSS.
