# Supplementary material for: Inequality in electricity consumption and economic growth: Evidence from a small area estimation study
Source: PLoS One. 2023 Jul 26;18(7):e0284055. doi: 10.1371/journal.pone.0284055 (PMC10370772; doi:10.1371/journal.pone.0284055)
Supplement: S3 Table — (DOCX) [file pone.0284055.s004.docx]

Table A.3: GLS regressions of log of monthly per capita kWh: Central Coast

| Explanatory variables | Coefficient | Std. Err. | t | \|Prob\|>t |
| --- | --- | --- | --- | --- |
| Intercept | 0.259 | 0.241 | 1.075 | 0.283 |
| Commune proportion of households having fridge | 0.558 | 0.165 | 3.376 | 0.001 |
| Having television (yes=1; no=0) | 0.455 | 0.047 | 9.800 | 0.000 |
| Commune proportion of households having television | 1.072 | 0.195 | 5.490 | 0.000 |
| Average household size of commune | -0.100 | 0.040 | -2.491 | 0.013 |
| Log of per capita living area | 0.413 | 0.025 | 16.762 | 0.000 |
| Proportion of household members with upper-secondary school and above | 0.338 | 0.054 | 6.266 | 0.000 |
| Commune proportion of households having unimproved latrine | -0.239 | 0.066 | -3.623 | 0.000 |
| Commune proportion of households having solid wall house | 0.387 | 0.112 | 3.463 | 0.001 |
| Urban * Commune proportion of households having desk telephone | -0.620 | 0.177 | -3.500 | 0.001 |
| Urban * Commune proportion of not working people | 1.846 | 0.371 | 4.983 | 0.000 |
| Number of observations | 2012 |  |  |  |
| R2-adjusted | 0.551 |  |  |  |
| Rho | 0.132 |  |  |  |

Notes: the estimation results are obtained from using data contained in the 2009 VPHC and the 2010 VHLSS.
