# Supplementary material for: Inequality in electricity consumption and economic growth: Evidence from a small area estimation study
Source: PLoS One. 2023 Jul 26;18(7):e0284055. doi: 10.1371/journal.pone.0284055 (PMC10370772; doi:10.1371/journal.pone.0284055)
Supplement: S4 Table — (DOCX) [file pone.0284055.s005.docx]

Table A.4: GLS regressions of log of monthly per capita kWh: Central Highlands

| Explanatory variables | Coefficient | Std. Err. | t | \|Prob\|>t |
| --- | --- | --- | --- | --- |
| Intercept | 0.383 | 0.278 | 1.374 | 0.170 |
| Commune proportion of households having television | 1.815 | 0.275 | 6.592 | 0.000 |
| Commune proportion of household head with primary school | -0.799 | 0.318 | -2.515 | 0.012 |
| Head with lower-secondary education | -0.749 | 0.355 | -2.114 | 0.035 |
| Ethnic minorities (yes=1; no=0) | -0.333 | 0.081 | -4.134 | 0.000 |
| Log of per capita living area | 0.500 | 0.041 | 12.094 | 0.000 |
| Urban * Kinh & Hoa | 0.236 | 0.083 | 2.845 | 0.005 |
| Urban * Proportion of households without primary school | -0.385 | 0.177 | -2.170 | 0.030 |
| Number of observations | 627 |  |  |  |
| R2-adjusted | 0.450 |  |  |  |
| Rho | 0.165 |  |  |  |

Notes: the estimation results are obtained from using data contained in the 2009 VPHC and the 2010 VHLSS.
