# Supplementary material for: Inequality in electricity consumption and economic growth: Evidence from a small area estimation study
Source: PLoS One. 2023 Jul 26;18(7):e0284055. doi: 10.1371/journal.pone.0284055 (PMC10370772; doi:10.1371/journal.pone.0284055)
Supplement: S5 Table — (DOCX) [file pone.0284055.s006.docx]

Table A.5: GLS regressions of log of monthly per capita kWh: South East

| Explanatory variables | Coefficient | Std. Err. | t | \|Prob\|>t |
| --- | --- | --- | --- | --- |
| Intercept | 1.715 | 0.231 | 7.409 | 0.000 |
| Commune proportion of households having fridge | 0.644 | 0.173 | 3.715 | 0.000 |
| Having motorbike (yes=1; no=0) | 0.385 | 0.081 | 4.749 | 0.000 |
| Log of per capita living area | 0.478 | 0.034 | 13.868 | 0.000 |
| Commune proportion of children | -1.908 | 0.730 | -2.612 | 0.009 |
| Proportion of household members with primary school | -0.188 | 0.091 | -2.071 | 0.039 |
| Urban | 0.157 | 0.083 | 1.889 | 0.050 |
| Having house with solid wall (yes=1; no=0) | 0.196 | 0.088 | 2.232 | 0.026 |
| Number of observations | 1085 |  |  |  |
| R2-adjusted | 0.294 |  |  |  |
| Rho | 0.059 |  |  |  |

Notes: the estimation results are obtained from using data contained in the 2009 VPHC and the 2010 VHLSS.
