# Supplementary material for: Inequality in electricity consumption and economic growth: Evidence from a small area estimation study
Source: PLoS One. 2023 Jul 26;18(7):e0284055. doi: 10.1371/journal.pone.0284055 (PMC10370772; doi:10.1371/journal.pone.0284055)
Supplement: S6 Table — (DOCX) [file pone.0284055.s007.docx]

Table A.6: GLS regressions of log of monthly per capita kWh: Mekong River Delta

| Explanatory variables | Coefficient | Std. Err. | t | \|Prob\|>t |
| --- | --- | --- | --- | --- |
| Intercept | 1.351 | 0.159 | 8.487 | 0.000 |
| Commune proportion of households having fridge | 1.351 | 0.200 | 6.770 | 0.000 |
| Having television (yes=1; no=0) | 0.730 | 0.085 | 8.636 | 0.000 |
| Ethnic minorities (yes=1; no=0) | -0.345 | 0.087 | -3.969 | 0.000 |
| Household size | -0.069 | 0.014 | -5.068 | 0.000 |
| Log of per capita living area | 0.354 | 0.034 | 10.274 | 0.000 |
| Proportion of households without primary school | -0.255 | 0.059 | -4.287 | 0.000 |
| Having house with solid roof (yes=1; no=0) | 0.433 | 0.111 | 3.910 | 0.000 |
| Having tap water (yes=1; no=0) | 0.117 | 0.048 | 2.414 | 0.016 |
| Urban * Household size | 0.033 | 0.014 | 2.312 | 0.021 |
| Number of observations | 1821 |  |  |  |
| R2-adjusted | 0.349 |  |  |  |
| Rho | 0.085 |  |  |  |

Notes: the estimation results are obtained from using data contained in the 2009 VPHC and the 2010 VHLSS.
