# Supplementary material for: Inequality in electricity consumption and economic growth: Evidence from a small area estimation study
Source: PLoS One. 2023 Jul 26;18(7):e0284055. doi: 10.1371/journal.pone.0284055 (PMC10370772; doi:10.1371/journal.pone.0284055)
Supplement: S7 Table — (DOCX) [file pone.0284055.s008.docx]

Table A.7: Provincial estimates of electricity consumption

| Provinces | Sampled households | % household without electricity | Per capita electricity consumption (kWh/month) | | Gini of electricity consumption | |
| --- | --- | --- | --- | --- | --- | --- |
|  |  |  | Mean | Std. Err. | Mean | Std. Err. |
| *Northern Mountain* |  |  |  |  |  |  |
| Ha Giang | 50329 | 34.59 | 8.61 | 1.16 | 0.611 | 0.025 |
| Cao Bang | 56518 | 26.17 | 11.93 | 1.54 | 0.576 | 0.025 |
| Bac Kan | 34412 | 18.10 | 13.37 | 1.56 | 0.504 | 0.024 |
| Tuyen Quang | 34849 | 6.78 | 17.64 | 2.74 | 0.483 | 0.027 |
| Lao Cai | 42673 | 22.56 | 13.93 | 2.33 | 0.605 | 0.026 |
| Dien Bien | 38480 | 33.47 | 9.57 | 1.46 | 0.649 | 0.029 |
| Lai Chau | 25207 | 50.32 | 7.28 | 1.16 | 0.630 | 0.030 |
| Son La | 53611 | 29.56 | 10.77 | 1.25 | 0.589 | 0.019 |
| Yen Bai | 43750 | 10.69 | 16.77 | 2.23 | 0.519 | 0.023 |
| Hoa Binh | 56998 | 4.16 | 16.19 | 2.08 | 0.510 | 0.028 |
| Thai Nguyen | 54397 | 1.89 | 22.78 | 3.10 | 0.435 | 0.026 |
| Lang Son | 54098 | 11.72 | 13.81 | 1.48 | 0.517 | 0.023 |
| Bac Giang | 61311 | 1.15 | 20.78 | 2.38 | 0.396 | 0.024 |
| Phu Tho | 78687 | 2.11 | 23.60 | 2.58 | 0.434 | 0.020 |
| *Red River Delta* |  |  |  |  |  |  |
| Ha Noi | 182387 | 0.09 | 47.69 | 1.94 | 0.395 | 0.012 |
| Quang Ninh | 66403 | 3.61 | 35.61 | 1.96 | 0.364 | 0.013 |
| Vinh Phuc | 44844 | 0.23 | 27.63 | 1.37 | 0.314 | 0.016 |
| Bac Ninh | 47679 | 0.08 | 29.62 | 1.48 | 0.323 | 0.014 |
| Hai Duong | 80448 | 0.19 | 25.53 | 1.36 | 0.326 | 0.012 |
| Hai Phong | 83700 | 0.23 | 36.72 | 1.90 | 0.372 | 0.015 |
| Hung Yên | 58977 | 0.30 | 25.58 | 1.30 | 0.314 | 0.012 |
| Thai Bình | 57653 | 0.20 | 22.14 | 1.30 | 0.316 | 0.016 |
| Ha Nam | 38936 | 0.19 | 23.11 | 1.45 | 0.309 | 0.016 |
| Nam Dinh | 68076 | 0.22 | 23.88 | 1.33 | 0.316 | 0.016 |
| Ninh Bình | 49884 | 0.35 | 24.36 | 1.43 | 0.331 | 0.017 |
| *Central Coast* |  |  |  |  |  |  |
| Thanh Hoa | 154616 | 3.16 | 17.76 | 0.86 | 0.377 | 0.013 |
| Nghe An | 117793 | 4.96 | 18.22 | 1.22 | 0.401 | 0.020 |
| Ha Tinh | 72800 | 0.76 | 17.60 | 0.93 | 0.366 | 0.015 |
| Quang Binh | 39057 | 3.29 | 18.95 | 1.46 | 0.399 | 0.020 |
| Quang Tri | 40966 | 2.69 | 18.28 | 1.31 | 0.388 | 0.015 |
| Thua Thiên Hue | 44048 | 1.51 | 22.47 | 1.84 | 0.359 | 0.019 |
| Da Nang | 40264 | 0.23 | 39.68 | 3.18 | 0.353 | 0.020 |
| Quang Nam | 83416 | 4.07 | 19.01 | 1.08 | 0.381 | 0.012 |
| Quang Ngãi | 69184 | 2.83 | 20.46 | 1.28 | 0.363 | 0.014 |
| Binh Dinh | 57182 | 1.02 | 24.26 | 1.64 | 0.349 | 0.021 |
| Phú Yên | 46480 | 1.55 | 21.38 | 1.45 | 0.362 | 0.021 |
| Khanh Hoa | 40005 | 2.41 | 26.29 | 2.31 | 0.381 | 0.019 |
| Ninh Thuan | 30575 | 2.17 | 19.62 | 1.72 | 0.368 | 0.019 |
| Binh Thuan | 51748 | 5.04 | 20.77 | 1.38 | 0.357 | 0.018 |
| *Central Highlands* |  |  |  |  |  |  |
| Kon Tum | 31082 | 4.76 | 17.18 | 3.12 | 0.421 | 0.029 |
| Gia Lai | 74845 | 6.60 | 17.77 | 2.33 | 0.414 | 0.023 |
| Dak Lak | 69869 | 6.97 | 19.57 | 2.65 | 0.400 | 0.025 |
| Dak Nong | 33905 | 16.60 | 16.60 | 2.23 | 0.428 | 0.030 |
| Lâm Dong | 60162 | 4.90 | 20.68 | 2.58 | 0.377 | 0.027 |
| *South East* |  |  |  |  |  |  |
| Binh Phuoc | 41766 | 10.68 | 27.52 | 1.87 | 0.368 | 0.018 |
| Tay Ninh | 53625 | 2.46 | 30.90 | 2.29 | 0.359 | 0.017 |
| Binh Duong | 42661 | 1.01 | 33.01 | 2.82 | 0.392 | 0.018 |
| Dong Nai | 67714 | 3.75 | 32.69 | 2.70 | 0.374 | 0.017 |
| Ba Ria - Vung Tau | 41954 | 0.97 | 38.10 | 3.21 | 0.381 | 0.018 |
| Ho Chí Minh | 143839 | 0.51 | 57.79 | 3.66 | 0.400 | 0.017 |
| *Mekong River Delta* |  |  |  |  |  |  |
| Long An | 75374 | 3.07 | 27.38 | 2.11 | 0.394 | 0.016 |
| Tien Giang | 60058 | 0.65 | 26.56 | 2.01 | 0.376 | 0.018 |
| Ben Tre | 54627 | 3.22 | 25.05 | 1.94 | 0.387 | 0.019 |
| Tra Vinh | 45984 | 8.92 | 18.90 | 1.45 | 0.438 | 0.020 |
| Vinh Long | 47094 | 4.00 | 25.24 | 2.06 | 0.396 | 0.019 |
| Dong Thap | 59947 | 3.05 | 21.29 | 1.40 | 0.397 | 0.015 |
| An Giang | 59962 | 7.71 | 21.84 | 1.55 | 0.432 | 0.017 |
| Kiên Giang | 69873 | 8.91 | 20.85 | 1.49 | 0.434 | 0.017 |
| Can Tho | 41039 | 1.94 | 30.84 | 2.68 | 0.428 | 0.022 |
| Hau Giang | 37052 | 4.52 | 21.12 | 1.70 | 0.391 | 0.018 |
| Soc Trang | 47056 | 8.18 | 18.12 | 1.50 | 0.450 | 0.019 |
| Bac Liêu | 34608 | 5.49 | 19.77 | 1.49 | 0.406 | 0.019 |
| Ca Mau | 45505 | 6.47 | 22.38 | 1.72 | 0.430 | 0.018 |

Notes: the estimation results are obtained from using data contained in the 2009 VPHC and the 2010 VHLSS.
