# Supplementary material for: Inequality in electricity consumption and economic growth: Evidence from a small area estimation study
Source: PLoS One. 2023 Jul 26;18(7):e0284055. doi: 10.1371/journal.pone.0284055 (PMC10370772; doi:10.1371/journal.pone.0284055)
Supplement: S8 Table — (DOCX) [file pone.0284055.s009.docx]

Table A.8. Regression of Gini index with control variables

| Explanatory variables | Dependent variable is Gini index of per capita kWh consumption | | | | Dependent variable is Gini index of per capita expenditure | |
| --- | --- | --- | --- | --- | --- | --- |
|  | OLS | Spatial regression | OLS | Spatial regression | OLS | Spatial regression |
| Log of per capita expenditure | -1.3656*** | -0.7972*** |  |  | -0.5116*** | -0.3033*** |
|  | (0.154) | (0.007) |  |  | (0.096) | (0.085) |
| Squared log of per capita expenditure | 0.0678*** | 0.0386*** |  |  | 0.0264*** | 0.0158*** |
|  | (0.008) | (0.001) |  |  | (0.005) | (0.005) |
| Log of monthly per capita kWh |  |  | -0.3379*** | -0.2557*** |  |  |
|  |  |  | (0.072) | (0.044) |  |  |
| Squared log of monthly per capita kWh |  |  | 0.0324** | 0.0236** |  |  |
|  |  |  | (0.014) | (0.009) |  |  |
| Log of population density | Reference |  |  |  |  |  |
|  |  |  |  |  |  |  |
| Share of urban population (in percent) | -0.1023*** | -0.1127*** | -0.2607*** | -0.2483*** | -0.0121*** | -0.0109** |
|  | (0.006) | (0.005) | (0.013) | (0.021) | (0.004) | (0.005) |
| Northern Mountains | -0.1043*** | -0.0946*** | -0.2707*** | -0.2402*** | -0.0228*** | -0.0202*** |
|  | (0.005) | (0.005) | (0.012) | (0.021) | (0.003) | (0.006) |
| Red River Delta | -0.0916*** | -0.0957*** | -0.2013*** | -0.1658*** | 0.0315*** | 0.0350*** |
|  | (0.006) | (0.008) | (0.015) | (0.025) | (0.005) | (0.007) |
| Central Coast | -0.0517*** | -0.0409*** | -0.0938*** | -0.0360* | 0.0132*** | 0.0255*** |
|  | (0.006) | (0.005) | (0.016) | (0.019) | (0.004) | (0.005) |
| Central Highlands | -0.0317*** | -0.0418*** | -0.0655*** | -0.0560*** | -0.0152*** | -0.0154*** |
|  | (0.006) | (0.004) | (0.013) | (0.018) | (0.003) | (0.005) |
| South East | -0.0110*** | -0.0113*** | -0.0282*** | -0.0351*** | -0.0051*** | -0.0077*** |
|  | (0.002) | (0.002) | (0.004) | (0.005) | (0.001) | (0.001) |
| Mekong River Delta | 0.0007*** | 0.0007*** | 0.0024*** | 0.0022*** | 0.0005*** | 0.0006*** |
|  | (0.000) | (0.000) | (0.000) | (0.000) | (0.000) | (0.000) |
| Lambda |  | 0.0016** |  | -0.0012* |  | 0.0012* |
|  |  | (0.001) |  | (0.001) |  | (0.001) |
| Rho |  | 0.2034*** |  | 0.1107*** |  | 0.1102*** |
|  |  | (0.014) |  | (0.004) |  | (0.004) |
| Constant | 7.1909*** | 4.4087 | -0.3747*** | -0.6357*** | 2.7127*** | 1.6614*** |
|  | (0.737) | (0.000) | (0.100) | (0.079) | (0.459) | (0.402) |
| Observations | 675 | 675 | 675 | 675 | 675 | 675 |
| R-squared | 0.786 |  | 0.801 |  | 0.550 |  |
| Robust standard errors in parentheses.  * significant at 10%; ** significant at 5%; *** significant at 1%.  Source: Estimation from the 2009 VPHC and the 2010 VHLSS. | | | | | | |
