# Supplementary material for: Inequality in electricity consumption and economic growth: Evidence from a small area estimation study
Source: PLoS One. 2023 Jul 26;18(7):e0284055. doi: 10.1371/journal.pone.0284055 (PMC10370772; doi:10.1371/journal.pone.0284055)
Supplement: S9 Table — (DOCX) [file pone.0284055.s010.docx]

Table A.9. Regression of log of Gini index

| Explanatory variables | Dependent variable is Log of Gini index of per capita kWh consumption | | | | Dependent variable is Log of Gini index of per capita expenditure | |
| --- | --- | --- | --- | --- | --- | --- |
|  | OLS | Spatial regression | OLS | Spatial regression | OLS | Spatial regression |
| Log of per capita expenditure | -4.8375*** | -6.0969*** |  |  | -3.3129*** | -4.1492*** |
|  | (0.5587) | (0.3796) |  |  | (0.3993) | (0.3340) |
| Squared log of per capita expenditure | 0.2415*** | 0.3204*** |  |  | 0.1739*** | 0.2266*** |
|  | (0.0292) | (0.0200) |  |  | (0.0210) | (0.0175) |
| Log of monthly per capita kWh |  |  | -0.6855*** | -0.7341*** |  |  |
|  |  |  | (0.0919) | (0.0469) |  |  |
| Squared log of monthly per capita kWh |  |  | 0.0912*** | 0.1311*** |  |  |
|  |  |  | (0.0151) | (0.0079) |  |  |
| Lambda |  | 0.0095*** |  | 0.0076*** |  | 0.0038*** |
|  |  | (0.0006) |  | (0.0004) |  | (0.0004) |
| Rho |  | 0.0799*** |  | 0.1014*** |  | 0.0967*** |
|  |  | (0.0025) |  | (0.0035) |  | (0.0023) |
| Constant | 23.1031*** | 28.2516*** | 0.1834 | 0.2033*** | 14.4536*** | 17.7879*** |
|  | (2.6692) | (1.8048) | (0.1379) | (0.0736) | (1.8987) | (1.5997) |
| Observations | 675 | 675 | 675 | 675 | 675 | 675 |
| R-squared | 0.3753 |  | 0.3632 |  | 0.1616 |  |
| Robust standard errors in parentheses.  * significant at 10%; ** significant at 5%; *** significant at 1%.  Source: Estimation from the 2009 VPHC and the 2010 VHLSS. | | | | | | |
