# Supplementary material for: Inequality in electricity consumption and economic growth: Evidence from a small area estimation study
Source: PLoS One. 2023 Jul 26;18(7):e0284055. doi: 10.1371/journal.pone.0284055 (PMC10370772; doi:10.1371/journal.pone.0284055)
Supplement: S10 Table — (DOCX) [file pone.0284055.s011.docx]

Table A.10. Regression of log of Gini index with control variables

| Explanatory variables | Dependent variable is Log of Gini index of per capita kWh consumption | | | | Dependent variable is Log of Gini index of per capita expenditure | |
| --- | --- | --- | --- | --- | --- | --- |
|  | OLS | Spatial regression | OLS | Spatial regression | OLS | Spatial regression |
| Log of per capita expenditure | -3.2284*** | -2.9216*** |  |  | -1.8177*** | -1.2787*** |
|  | (0.365) | (0.311) |  |  | (0.326) | (0.319) |
| Squared log of per capita expenditure | 0.1609*** | 0.1475*** |  |  | 0.0945*** | 0.0674*** |
|  | (0.019) | (0.017) |  |  | (0.017) | (0.017) |
| Log of monthly per capita kWh |  |  | -0.3379*** | -0.2557*** |  |  |
|  |  |  | (0.072) | (0.044) |  |  |
| Squared log of monthly per capita kWh |  |  | 0.0324** | 0.0236** |  |  |
|  |  |  | (0.014) | (0.009) |  |  |
| Log of population density |  |  |  |  |  |  |
|  |  |  |  |  |  |  |
| Share of urban population (in percent) | -0.2726*** | -0.2524*** | -0.2607*** | -0.2483*** | -0.0436*** | -0.0374** |
|  | (0.013) | (0.020) | (0.013) | (0.021) | (0.012) | (0.019) |
| Northern Mountains | -0.2626*** | -0.2238*** | -0.2707*** | -0.2402*** | -0.0843*** | -0.0761*** |
|  | (0.012) | (0.021) | (0.012) | (0.021) | (0.011) | (0.020) |
| Red River Delta | -0.2191*** | -0.1667*** | -0.2013*** | -0.1658*** | 0.0999*** | 0.1129*** |
|  | (0.015) | (0.025) | (0.015) | (0.025) | (0.017) | (0.023) |
| Central Coast | -0.1189*** | -0.0599*** | -0.0938*** | -0.0360* | 0.0440*** | 0.0903*** |
|  | (0.015) | (0.018) | (0.016) | (0.019) | (0.014) | (0.016) |
| Central Highlands | -0.0619*** | -0.0671*** | -0.0655*** | -0.0560*** | -0.0544*** | -0.0583*** |
|  | (0.013) | (0.019) | (0.013) | (0.018) | (0.011) | (0.017) |
| Southeast | -0.0330*** | -0.0355*** | -0.0282*** | -0.0351*** | -0.0210*** | -0.0274*** |
|  | (0.004) | (0.005) | (0.004) | (0.005) | (0.004) | (0.005) |
| Mekong River Delta | 0.0020*** | 0.0018*** | 0.0024*** | 0.0022*** | 0.0019*** | 0.0020*** |
|  | (0.000) | (0.000) | (0.000) | (0.000) | (0.000) | (0.000) |
| Lambda |  | 0.0003 |  | -0.0012* |  | -0.0005 |
|  |  | (0.001) |  | (0.001) |  | (0.001) |
| Rho |  | 0.1083*** |  | 0.1107*** |  | 0.1097*** |
|  |  | (0.004) |  | (0.004) |  | (0.005) |
| Constant | 15.0201*** | 13.3001*** | -0.3747*** | -0.6357*** | 7.2902*** | 4.5517*** |
|  | (1.732) | (1.473) | (0.100) | (0.079) | (1.555) | (1.512) |
| Observations | 675 | 675 | 675 | 675 | 675 | 675 |
| R-squared | 0.798 |  | 0.801 |  | 0.560 |  |
| Robust standard errors in parentheses.  * Significant at 10%; ** significant at 5%; *** significant at 1%.  Source: Estimation from the 2009 VPHC and the 2010 VHLSS. | | | | | | |
