# Supplementary material for: Inequality in electricity consumption and economic growth: Evidence from a small area estimation study
Source: PLoS One. 2023 Jul 26;18(7):e0284055. doi: 10.1371/journal.pone.0284055 (PMC10370772; doi:10.1371/journal.pone.0284055)
Supplement: S1 Fig — (DOCX) [file pone.0284055.s012.docx]

Figure A.1: Gini index of monthly kWh and Gini index of per capita expenditure

| Provinces | Districts |
| --- | --- |
|  |  |

Source: authors’ estimation from the 2009 VPHC and the 2010 VHLSS.
