# Supplementary material for: Inequality in electricity consumption and economic growth: Evidence from a small area estimation study
Source: PLoS One. 2023 Jul 26;18(7):e0284055. doi: 10.1371/journal.pone.0284055 (PMC10370772; doi:10.1371/journal.pone.0284055)
Supplement: Attachment — Submitted filename: Responses to reviewers_R1.docx [file pone.0284055.s013.docx]

**Note on revision**

Manuscript "Inequality in Electricity Consumption and Economic Growth: Evidence from a Small Area Estimation Study" (manuscript No. PONE-D-22-30320)

Dear Dr. Carlos Alberto Zúniga-González,

Academic Editor, PLOS ONE

We would like to thank you very much for spending time providing detailed comments and for giving us a chance to revise and re-submit our paper "Inequality in Electricity Consumption and Economic Growth: Evidence from a Small Area Estimation Study" (manuscript No. PONE-D-22-30320) to *PLOS ONE*. You and the two reviewers provided useful comments on our studies. During the past month, we have made revisions and extensions of the paper to address all the questions and issues raised in the referee reports. Please see below our response to each of the specific comments from the referees.

Thank you again for these comments and for offering us the R&R opportunity at *PLOS ONE.*

Best regards,

Authors

**Comments from editors**

Dear author Tuyen Tran, I have read your manuscript and it is interesting, but I suggest you must add a discussion section (regarding Gini and model used), where you compare your results with other authors, y section 3 or before a add a bit more literature review where you say the author that use the method of analysis. also, I would like to suggest adding more reference may be 40.

*Authors: Thank you very much for this useful comment. Following you suggestion, we added more discussion comparing our findings with other studies. We also increased the number of references to 40. We added the following paragraphs.*

*“Similar findings were observed by Hasan and Mozumder (2017) who found that a U-shaped relationship exists between income and electricity consumption in Bangladesh. However, our result is partly inconsistent with some studies in other developing countries. For instance, Ma et al. (2021), find in rural China that energy inequality decreases with higher levels of energy consumption. while the distribution of electricity consumption was quite equal among income group in rural China (Wu, Zheng, & Wei, 2017). Also, electricity consumption inequality was found to increase with income levels in Indonesia (Dwi Cahyani, Nachrowi, Hartono, & Widyawati, 2020).”*

Following your recommendation, we also expanded the literature review and included some new references to the methods used in Section 3.1

*Small-area estimation has gained a lot of attention in recent years due to the growing need for accurate small-area estimates from both the public and business sectors (Corral, Molina, Cojocaru, & Segovia, 2022; Perseh et al., 2023; Rahman, 2008). Using the small area estimation method, Perseh et al. (2023) estimated indquality indicators in diabetes care and health outcomes relevant to diabetes management in almost all districts of Iran. Reames (2016) predicted the mean census block group home heating energy usage intensity (EUI), an energy efficiency proxy, in Kansas City, Missouri, using small-area estimate approaches. Using this method, numerous studies have predicted poverty estimates at the different disaggregation levels in Vietnam ( Nguyen, 2011), Kenya (Christiaensen, Lanjouw, Luoto, & Stifel, 2012), Spain (Esteban, Morales, Pérez, & Santamaría, 2012) and India (Chandra, Aditya, & Sud, 2018).”*

**Reviewer 1**
The article adequately presents the methodology used to process the data necessary to examine the relationship between social inequity and electricity consumption. In my opinion, methodological support has been presented for the results shown; especially in those related to U-shaped relation between the inequality of electricity consumption and per capita household expenditure in Vietnamese communities.

*Authors: Thank you very much for your comments.*

**Reviewer 2**
The research topic is interesting. This research is very well designed and developed.
However, I have the following comments about this study:
1. The description and use of the estimation method is not clear. The 2010 VHLSS and the 2009 VPHC are two different data sets. How to merger these data to estimate?...

*Authors: Thank you very much. Actually, we do not really merge the two data sets. Our main objective is to estimate the relation between inequality and mean electricity consumption at the district and province levels. The 2010 VHLSS contains data about not only expenditure on electricity but also the number of kilowatt hours (kWh) that households consumed in the last month. However, the 2010 VHLSS covered 9,399 households. It is just a sampled survey, which is not representative for provinces and districts. On the other hand, the 2009 VPHC has a large coverage of households, and it is representative at the provincial and district levels, but it does not contain data on electricity consumption of households. We first use the 2010 VHLSS to construct a model of electricity consumption as a function of household and community variables that are are available in both the 2010 VHLSS and the 2009 VHPC. Then, the parameter estimates from this model are applied to the 2009 VHPC to predict electricity consumption of all households in the population. These household-level data allow use to estimate the mean and inequality of electricity consumption for provincial and districts.*

In the revised manuscript, we added a paragraph in the Introduction section to note the data combination.

2. In table 2, are the estimated results robust or not?

*Authors: Thank you very much for this useful comment. Following you suggestion, we conducted additional robustness analyses. Firstly, we try both OLS and spatial regressions, which give quite similar results. Secondly, we examine whether the estimates are sensitive to inclusion of additional control variables. In Table A.8 in the Appendix, we include additional control variables including regional dummies, log of population density and the share of urban population of districts. Overall, the model with control variables gives similar results as the model without control variables in Table 2. In this study, we aim to examine the change in electricity inequality across the economic development level instead of estimating the causal effect of the economic development. Thus, we will use the result from regression without control variables for interpretation. Thirdly, in addition to the Gini index, we use log of the Gini index as the dependent variable. The results, which are reported in Tables A.9 and A.10 in the Appendix, show similar findings as those in Table 2.*
