# Supplementary material for: Inequality in electricity consumption and economic growth: Evidence from a small area estimation study
Source: PLoS One. 2023 Jul 26;18(7):e0284055. doi: 10.1371/journal.pone.0284055 (PMC10370772; doi:10.1371/journal.pone.0284055)
Supplement: Attachment — Submitted filename: Authors_responses_R2.docx [file pone.0284055.s014.docx]

**Note on revision**

Manuscript "Inequality in Electricity Consumption and Economic Growth: Evidence from a Small Area Estimation Study" (PONE-D-22-30320R1)

Dear Dr. Carlos Alberto Zúniga-González,

Academic Editor, PLOS ONE

We would like to thank you very much for spending time providing detailed comments and for giving us a chance to revise and re-submit our paper "Inequality in Electricity Consumption and Economic Growth: Evidence from a Small Area Estimation Study" (manuscript No. PONE-D-22-30320R1) to *PLOS ONE*. We have made revisions and address all the questions and issues raised by the reviewer #2 . Please see below our response to each of the specific comments from the reviewer #2

Thank you again for these comments and for offering us the R&R opportunity at *PLOS ONE.*

Best regards,

Authors

Reviewer #2: From 2010 to now, the Government has invested in the energy sector for mountainous areas. The problem of energy poverty in Vietnam has also been improved significantly. So, I want the authors to further clarify the policy implications associated with the Vietnamese context.

Authors’ responses:

Thank you so much for your helpful comments. Following your suggestion, we have addressed this implicatin in the revision as follows:

Findings from our study suggest the provision of an electricity subsidy for low-income households, especially those in poor areas. The Vietnamese government has invested in the power sector for rural and mountainous areas since 2010; this has significantly improved the access to the electricity grid for a huge number of households (Minh and Nguyen, 2021). Since 2011, the government of Vietnam has been providing cash subsidies for electricity consumption to poor households (Government of Vietnam, 2011). The issue of energy poverty has been significantly reduced, and inequalities in electricity consumption also declined during the 2008–2018 period (Minh et al., 2021). However, the energy cost has recently increased, becoming a growing burden for low-income households (Nguyen, Nguyen, Hoang, Wilson, & Managi, 2019). Poor households face relatively high costs of energy, which can limit their affordability of other goods and consequently reduce their well-being (Nguyen et al., 2019). Thus, electricity-subsidy policies for poor households should be further strengthened and implemented.
